# 20.1 DISSECTING THE FUNCTIONAL CONSEQUENCES OF RECIPROCAL GENOMIC DISORDERS

**DOI:** 10.1093/schbul/sby014.080

**Published:** 2018-04-01

**Authors:** Michael Talkowski

**Affiliations:** Massachusetts General Hospital, Harvard Medical School

## Abstract

**Background:**

Reciprocal genomic disorders (RGDs) represent a unique class of recurrent genomic variation that offer insight into highly dosage sensitive regions of the morbid human genome. However, the genomic architecture mediating RGDs, namely non-allelic homologous recombination (NAHR) of flanking segmental duplications, has rendered these genomic segments recalcitrant to conventional model studies. We recently developed a novel CRISPR method that leverages the homology of segmental duplications and efficiently generates large microdeletions and microduplications that mimic NAHR in humans, including ablation or duplication of one copy equivalent of the segmental duplications. Here, we explore the functional consequences of 16p11.2 RGD in iPS derived neuronal models and across mouse tissues.

**Methods:**

We generated CRISPR-engineered 16p11.2 RGD models against an isogenic iPSC background and performed transcriptome profiling in iPSC-derived neural stem cells (NSCs) and induced neurons (iN) (n = 10 isogenic deletions, 10 duplications, 6 controls). We then integrated these data with RNAseq from 306 libraries from multiple tissues in 70 mouse models of reciprocal deletion and duplication of the syntenic 7qf3 region (cortex, striatum, cerebellum, liver, white fat, brown fat in 16 mice; and replication from cortex, striatum, cerebellum in 54 mice).

**Results:**

In ongoing analyses, weighted-gene correlation network analysis (WGCNA) identified co-expression modules that were significantly enriched for 16p11.2 genes, evolutionarily constrained genes, genes robustly associated with autism spectrum disorder (ASD; TADA q < 0.1) and developmental disorders (DDD). Pathway analyses within modules discovered enrichment of genes critical to synaptic formation and neural connectivity as well as the protocadherin gene family. Network analyses specific to brain tissues within modules further identified a convergence on highly connected, or ‘hub’ genes, on Wnt signaling, including Ctnnb1 and Ctnnd1. The module was also again enriched for ASD loci (TADA, FDR < 0.1), constrained genes (ExAC, pLI ≥ 0.9) and brain specific genes from the Human Protein Atlas.

**Discussion:**

These studies suggest the functional consequences of 16p11.2 RGD across models converge on transcriptional signatures associated with critical neurodevelopmental pathways and individual genes implicated in a spectrum of developmental and neuropsychiatric disorders.

